# Glucocorticoids induce osteonecrosis of the femoral head through the Hippo signaling pathway

**DOI:** 10.1515/biol-2021-0102

**Published:** 2021-10-26

**Authors:** Yugang Li, Zechuan Xu, Shan Chang

**Affiliations:** Department of Orthopedics, The First Affiliated Hospital of Chengdu Medical College, No. 278 Baoguang Avenue, Xindu District, Chengdu 610500, Sichuan, People’s Republic of China; Department of Orthopedics, The Second Affiliated Hospital of Chengdu Medical College (Nuclear Industry 416 Hospital), Chengdu 610057, Sichuan, People’s Republic of China

**Keywords:** BMSC, GC, ONFH, hippo signaling pathway

## Abstract

Osteonecrosis of the femoral head (ONFH) induced by glucocorticoids (GCs) has been considered to be associated with the dysfunction of bone marrow mesenchymal stem cells (BMSCs). Studies have reported that GCs can regulate the normal differentiation of BMSCs. However, the exact mechanism of this regulation remains unclear. In this study, we used methylprednisolone (MPS) to induce BMSCs, and then found that the Hippo signaling pathway was upregulated in a dose-dependent manner compared to that in the control group. In addition, the osteogenic ability of BMSCs was decreased, as evaluated by Alizarin Red S staining analysis and alkaline phosphatase activity assays, accompanied by the downregulated expression of Runx2, osteopontin, and osteocalcin. Additionally, the adipogenic capacity of BMSCs under the MPS conditions was increased, as identified by Oil Red O staining with upregulated triglyceride and PPARγ expression. Moreover, suppression by knockdown of MST1 was found to attenuate the Hippo signaling pathway and adipogenic differentiation, while enhancing osteogenic differentiation. In conclusion, our findings revealed that the Hippo signaling pathway was involved in GC-ONFH by affecting the osteogenic and adipogenic differentiation capacities of BMSCs. Our study could provide a basis for further investigation of the specific function of the Hippo pathway in ONFH.

## 1 Introduction

Osteonecrosis of the femoral head (ONFH), which mostly occurs following overdose glucocorticoid (GC) therapy, remains the most serious side effect of long-term or excessive steroid therapy [1]. ONFH is mainly due to insufficiency or interruption of the blood supply of the femoral head, resulting in apoptosis and necrosis of the cells. Epidemiological investigations in China have found that hormonal osteonecrosis accounts for approximately 24% of cases, especially in lupus erythematosus and rheumatoid arthritis patients requiring hormone therapy [2]. When ONFH is not properly treated, total hip arthroplasty is the only optional treatment. Unfortunately, most patients who suffer ONFH often need multiple operations over their lifetime. Consequently, the development of new targets for OHFH has become a primary current research topic.

A previous study demonstrated that bone marrow mesenchymal stem cells (BMSCs) play vital roles in bone formation [3]. BMSCs have been reported to be involved in balancing osteogenic and adipogenic differentiation, which may lead to osteoporosis [4]. Thus, dysregulation of BMSCs may result in orthopedic disorders [5]. In this study, we hypothesized that BMSCs also play important roles in the pathogenesis of GC-ONFH through abnormal directional differentiation.

The Hippo pathway is a kinase chain composed of protein kinases and transcription factors, which are also involved in regulating the cellular proliferation, differentiation, and maintenance of stem cells [6,7]. A previous study reported that the Hippo signaling pathway is involved in osteogenic differentiation though a transcriptional coactivator with a PDZ-binding motif (TAZ) [8]. Another study also showed that Hippo/TAZ was confirmed to promote alkaline phosphatase (ALP) activity, while the mineralized nodules increased significantly in human periodontal ligament stem cells [9]. In addition, the Hippo signaling pathway can regulate the adipogenic differentiation regulator peroxisome proliferator-activated receptor γ (PPARγ) and inhibit its downstream gene transcription [10]. Dong and Li found that activation of the Hippo signaling pathway could enhance the adipogenic capacity of mouse BMSCs *in vitro*, in which LATS2 played an important regulatory role [11]. Given that the Hippo pathway is associated with the processes of osteogenic differentiation and adipogenic differentiation, it is necessary to further explore whether the Hippo pathway may be the key mechanism in the regulation of GC-ONFH [12,13].

In this study, we demonstrated the role of the Hippo signaling pathway in regulating the adipo-osteogenic balance in MPS-induced BMSCs. The relationship between the Hippo signaling pathway and BMSCs treated with MPS was assessed, and the expressions of mammalian sterile 20-like kinase 1 (MST1), p-MST1, large tumor suppressor 1 and 2 (LATS1/2), p-LATS1/2, Yes-associated protein 1 (YAP), p-YAP, transcriptional coactivator with PDZ-binding motif (TAZ), and p-TAZ were examined by western blot. The osteogenic differentiation of BMSCs was analyzed by Alizarin Red S staining analysis and ALP activity assay, while the adipogenic differentiation ability was tested by Oil Red O staining and triglyceride (TG) analysis. We also observed the expression of PPARγ, runt-related gene 2 (Runx2), osteopontin (OPN), and osteocalcin (OCN) detected by qRT-PCR after methylprednisolone (MPS) treatment. Moreover, we further explored the mechanism of the Hippo signaling pathway in BMSCs treated with MPS after MST1 knockdown. The results showed that the Hippo signaling pathway plays an important role in the adipo-osteogenesis balance of BMSCs. Collectively, Hippo promoted the adipogenic differentiation of BMSCs and inhibited osteogenic differentiation under MPS conditions. These results also strongly suggest that the Hippo signaling pathway is an effective method to study human GC-ONFH.

## 2 Materials and methods

### 2.1 Cell culture

BMSCs were purchased from Guangzhou Cyagen Inc. (OriCell^®^, Article No: RASMX-01001) and cultured in low-glucose DMEM (Gibco, Germany) with 10% fetal bovine serum (FBS; Gibco, Germany) and 1% penicillin–streptomycin. The BMSCs were passaged upon reaching 80% confluence.

### 2.2 Flow cytometry

BMSCs were grown in 6-hole plate, digested with 0.25% trypsin, and centrifuged at 4°C (1,000 rpm) for 5 min. Then, the cells were collected and washed three times in PBS and the associated flow cell antibodies (CD44, CD45, and CD90) were added for 0.5 h at 4°C. Ultimately, after washing two times with PBS, the surface markers of the hematopoietic cells CD44, CD45, and CD90 were examined by flow cytometry (BD Company, America) according to the manufacturer’s protocol [11].

### 2.3 Cell treatment

BMSCs were continuously treated with MPS (Pfizer Pharmaceutical, China) at different concentrations (0, 10^−9^, 10^−7^, 10^−5^ mol/L) after the density of BMSCs reached 80% according to the CCK-8 assay. BMSCs were cultured in osteogenic differentiation medium or adipogenic differentiation medium and were treated with different concentrations of MPS (0, 10^−9^, 10^−7^, 10^−5^ mol/L) for 21 days for differentiation correlation experiments. To investigate the effects of MST1-specific siRNA (MST1-siRNA), BMSCs were treated with 10^−7^ mol/L MPS in osteogenic differentiation medium or adipogenic differentiation medium for 21 days.

### 2.4 Cell counting kit-8 (CCK-8) assay

The effect of MPS on the proliferation of BMSCs was determined using a CCK-8 kit (Beyotime, China). Briefly, the treated cells were cultivated in a 96-well plate, with 3 × 10^3^ cells/well. Ten microliters of CCK-8 reagent were added at different time points (days 0, 1, 2, 3, and 4) and tested at 450 nm with a microplate reader (Thermo Fisher, America).

### 2.5 Alizarin red S staining analysis (calcium nodule staining)

The cells were washed with PBS 1−2 times, fixed for 10 min with 95% ethanol, and rewashed 1−2 times using PBS. The cells were covered with 0.1% Alizarin Red S solution for 10 min. Finally, the cells were observed under an inverted light microscope (Olympus, Japan).

### 2.6 ALP activity assay

ALP activity was examined using an ALP activity kit (Beyotime, China) according to the manufacturer’s protocol. Cells were grown in 96-well plates (1 × 10^4^ cell/mL) and cultured after 14 days containing 10% fetal bovine serum for the ALP activity assay. After the operation, the mixture was lysed and incubated in 37°C light avoidance solution for 30 min, and 100 μL of reaction termination liquid was added to each hole to terminate the reaction. The absorbance was determined at 405 nm with enzyme and labeling instrument (Thermo Fisher, America).

### 2.7 Oil red O staining

The cells were washed twice and fixed with PBS solution and 4% paraformaldehyde for 30 min. Then, the cells were washed twice with PBS solution, stained with Oil Red O, and stained at room temperature for 30 min. After washing the cells twice, they were observed and photographed by ordinary optical microscopy (Olympus, Japan).

### 2.8 Determination of TG

The cells in the 6-well plate were digested with 0.25% trypsin, the supernatant was discarded, and the cells were precipitated. After washing with PBS twice, 1% TritonX-100 was added for 30 min, the mixture was mixed well at 37°C for 10 min, and the absorbance was measured at 510 nm by enzyme labeling instrument (Thermo Fisher, USA).

### 2.9 qRT-PCR

TRIzol reagent (Invitrogen, USA) was used to extract total RNA according to the manufacturer’s instructions. Then cDNA was synthesized by an PrimeScript RT Master Mix kit (TaKaRa Biotechnology, China). qRT-PCR was performed using SYBR Green Master Mix (Roche, USA). RNA expressions levels were analyzed using the 2^−ΔΔCt^-method. The cloned sequences were constructed by RiboBio (Guangzhou, China). The primers used are described in Table 1.

**Table 1:** Real-time PCR gene markers

Gene markers	Forward	Reverse
Runx	TTCAACGATCTGAGATTTGTGGG	GGATGAGGAATGCGCCCTA
OPN	CTGGCAGCTCAGGGAGAAG	TTCTGTGGCGCAAGGAGATT
OCN	GGGCTCCAAGTCCATTGTT	ACCCGAATGTTGAGCGAGAG
PPAPγ	CCCTTTACCACGGTTGATTTC	CTTCAATCGGATGGTTCTTCG
GAPDH	CCCAGAAGACTGTGGATGG	CACATTGGGGGTAGGAACAC

### 2.10 Western blot

In brief, the cells were washed with PBS three times and lysed and denatured in RIPA lysate (Beyotime Biotechnology, China), with protease inhibitor for 30 min. The protein samples were separated by SDS-PAGE and transferred to PVDF membranes (Millipore, USA). The membrane was blocked with TBST (T1085-500, Solarbio) containing 5% skimmed milk for 2 h and then incubated with primary antibodies including: anti-MST1 (0.2 µg/mL, ab245826, Abcam), anti-p-MST1 (1/500, ab79199, Abcam), anti-LATS1/2 (1/100, orb193134, Biobyt), anti-p-LATS1/2 (1/100, ab111344, Abcam), anti-YAP (1/5,000, ab52771, Abcam), anti-p-YAP (1/10,000, ab76252, Abcam), anti-TAZ (1 μg/mL, ab84927, Abcam), anti-p-TAZ (1/200, sc17610, Santa Cruz), and anti-GAPDH (1/2,500, ab9485, Abcam) overnight at 4°C. After being washed with PBS twice, HRP-conjugated secondary antibodies (1:5,000, Abcam) were incubated with the membranes at room temperature for 1 h. The diluent for secondary antibodies was TBST buffer. The membranes were detected using an ECL western blot kit (K820500, Biovision Inc., USA) and analyzed by ImageJ.

### 2.11 Transfection assay

MST1-siRNA was obtained from Thermo Fisher Company (GenePharma, China). The target sequence of MST1-siRNA was 5′-CCAUGACUGAUGGAGCCAATT-3′. MST1-siRNA was then transfected into BMSCs with Lipofectamine^®^RNAiMAX reagent following the manufacturer’s protocol. Forty-eight hours after transfection, the cells were collected for the following studies.

### 2.12 Statistical analysis

All data were analyzed by Graphpad 6.0 and are presented as mean ± SD. Statistical analysis was performed with one-way analysis of variance (ANOVA). *P* < 0.05 was regarded as statistically significant. The experiments were repeated three times.

## 3 Results

### 3.1 BMSC surface markers examination

**Figure 1:**
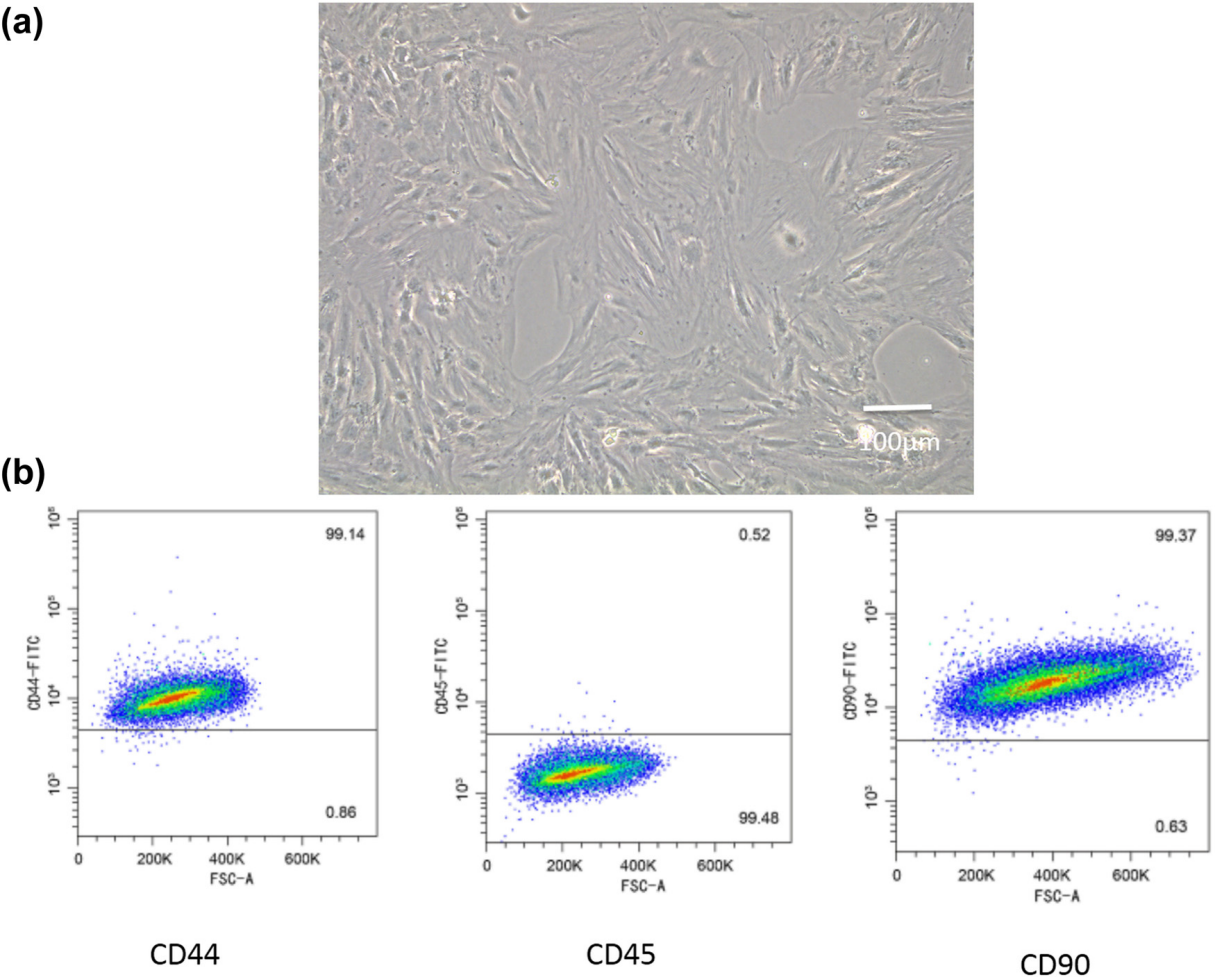
BMSC surface markers examination. (a) The BMSCs had a typical long-spindle-like appearance. (b) CD44 and CD90 in BMSCs were significantly increased compared with CD45.

Our study used commercially available BMSCs. After 3 days of culture, the cells were of a typical long-spindle-like appearance (Figure 1a). The results of flow cytometry showed that the expression levels of CD44 and CD90, characteristic markers of bone marrow-derived mesenchymal stem cells, were significantly increased (99.14 and 99.37%, respectively) compared with that of CD45 (0.52%) (Figure 1b).

### 3.2 The Hippo signaling pathway in BMSCs was mediated by MPS.

**Figure 2:**
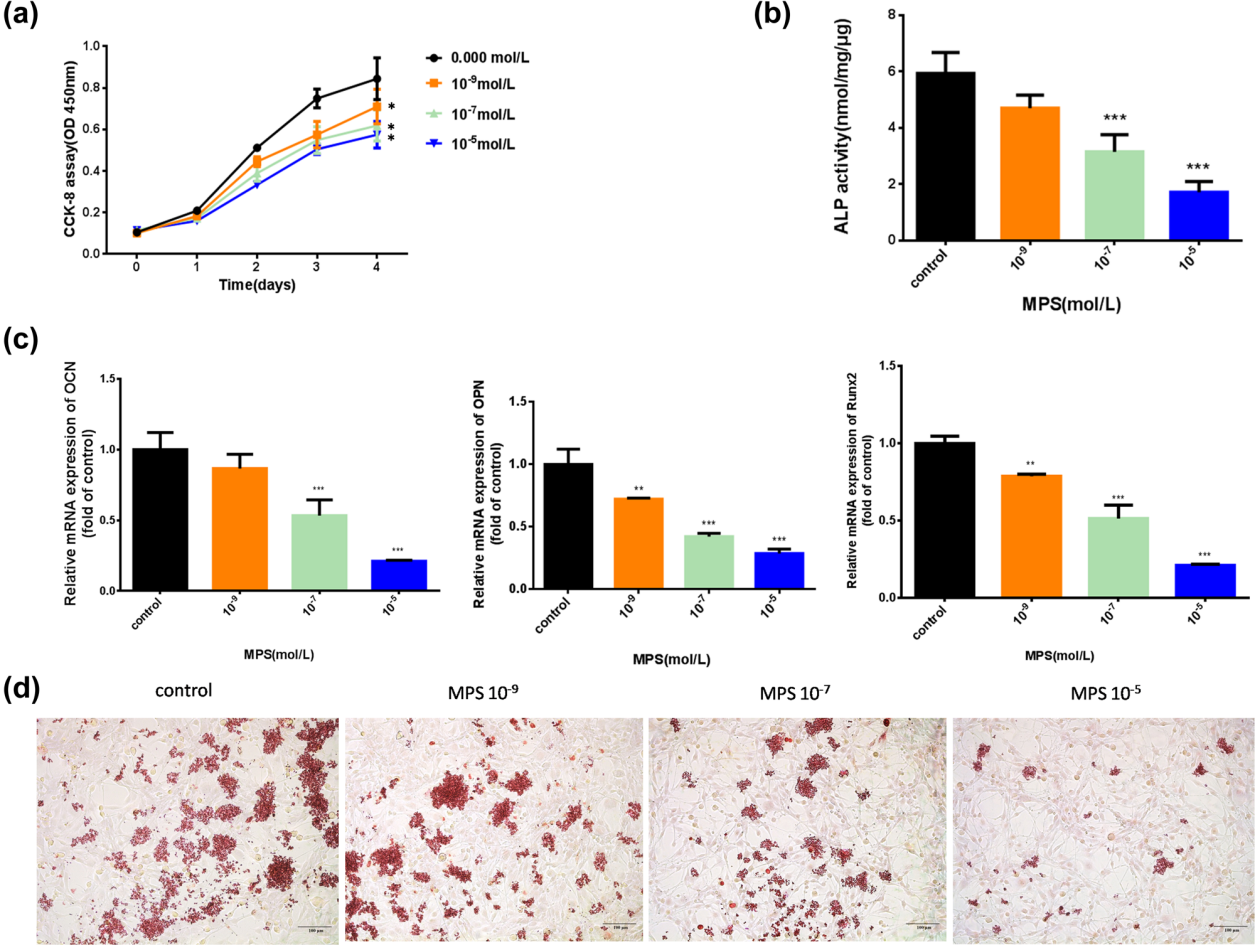
MPS inhibited BMSC proliferation and suppressed osteogenesis. (a) The viability of BMSCs under MPS conditions was evaluated by CCK-8. (b) The ALP activity under MPS conditions was detected by ALP activity assay. (c) The mRNA expressions levels of OCN, OPN, and Runx2 in BMSCs treated with MPS were evaluated by qRT-PCR. (d) The calcium nodule form in BMSCs was detected by Alizarin Red S staining analysis. *p < 0.05, **p < 0.01, ***p < 0.001. The experiment was repeated three times.

Next, we identified the mechanism of the Hippo signaling pathway in MPS-induced BMSCs. The western blot results showed that MPS significantly increased the expression levels of p-MST1, p-LATS1/2, p-YAP, and p-TAZ in a dose-dependent manner compared to the control, indicating that MPS could activate the Hippo signaling pathway (Figure 2).

### 3.3 MPS inhibited BMSC proliferation and suppressed osteogenesis.

**Figure 3:**
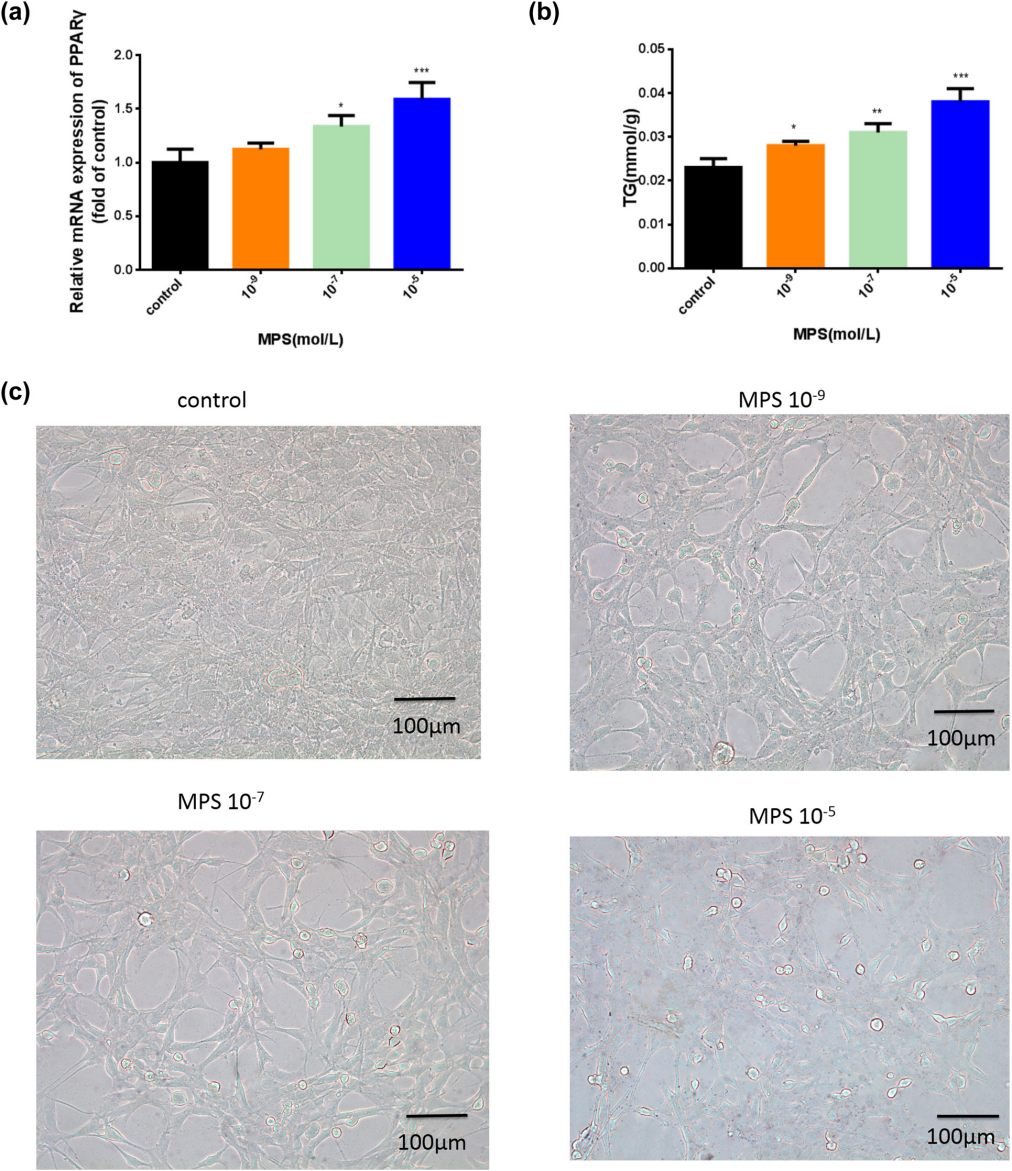
MPS promoted BMSC adipogenesis. (a) The mRNA expression of PPARγ was evaluated by qRT-PCR. (b) The content of TG was measured in BMSCs. (c) Oil droplets in BMSCs were observed using Oil Red O staining (×100). *p < 0.05, **p < 0.01, ***p < 0.001. The experiment was repeated three times.

To investigate the function of MPS in BMSCs, we verified the effects of different concentrations of MPS (10^−9^, 10^−7^, 10^−5^ mol/L) on BMSCs in the following experiments. The CCK-8 results showed that the proliferation of BMSCs was blocked upon stimulation with MPS, especially on the fourth day after MPS treatment (Figure 3a). In addition, to investigate the osteogenic effects of different concentrations of MPS, BMSCs were cultured in osteogenic induction medium with MPS treatment. The ALP activity assay results showed that MPS treatment inhibited the activity of ALP, especially at concentrations of 10^−9^ and 10^−7^ (Figure 3b). qRT-PCR results showed that MPS decreased the expressions levels of Runx2, OPN, and OCN, especially at concentrations of 10^−9^ and 10^−7^ MPS (Figure 3c). The Alizarin Red S staining analysis revealed that calcium in the control group had a darker staining than that in MPS-treated groups; meanwhile, with the increase of MPS concentration, the Alizarin Red S staining gradually decreased, suggesting that MPS could reduce the osteogenic differentiation capacity of BMSCs (Figure 3d).

### 3.4 MPS promoted BMSC adipogenesis.

**Figure 4:**
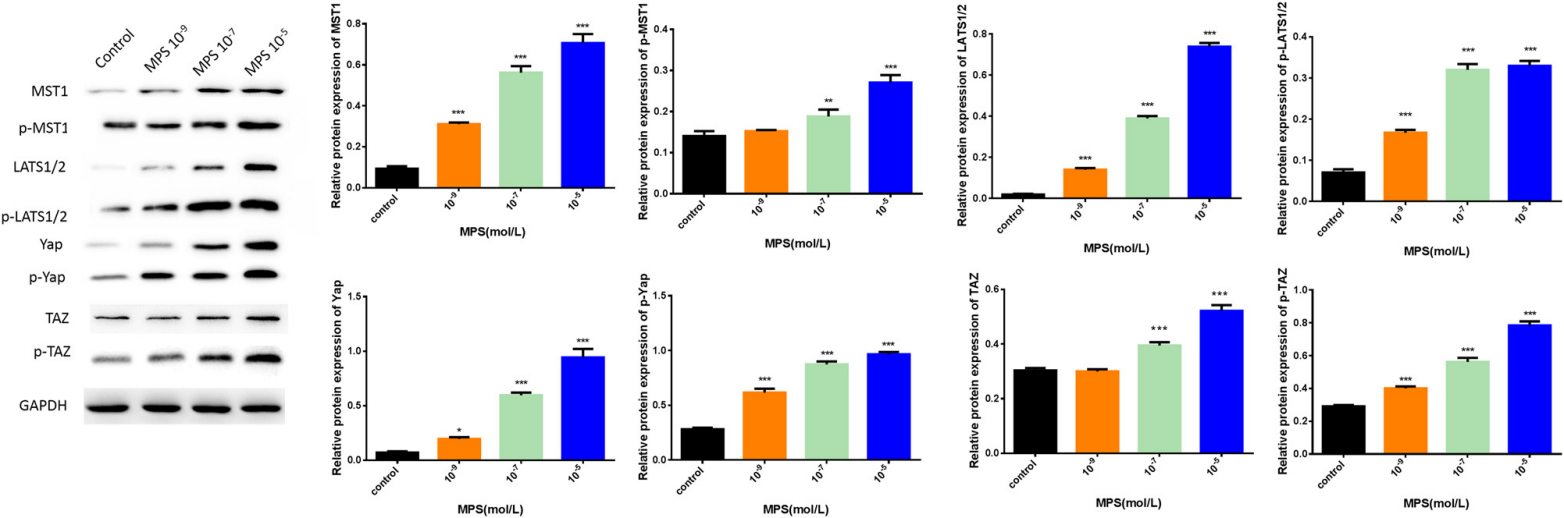
The Hippo signaling pathway in BMSCs was mediated by MPS. The protein expression levels of MST1, p-MST1, Yap, p-Yap, LATS1/2, p-LATS1/2, TAZ, and p-TAZ were detected by western blot. *p < 0.05, **p < 0.01, ***p < 0.001. The experiment was repeated three times.

The adipogenic differentiation of BMSCs was then carried out under MPS treatment. After 21 days of adipogenic induction, qRT-PCR results showed that PPARγ expression was decreased with increasing MPS concentration (Figure 4a). The TG content of BMSCs was increased (Figure 4b) and the number of droplets was increased using Oil Red O staining under MPS conditions (Figure 4c).

### 3.5 Evaluation of the effects of MST1-siRNA on MPS-treated BMSCs.

**Figure 5:**
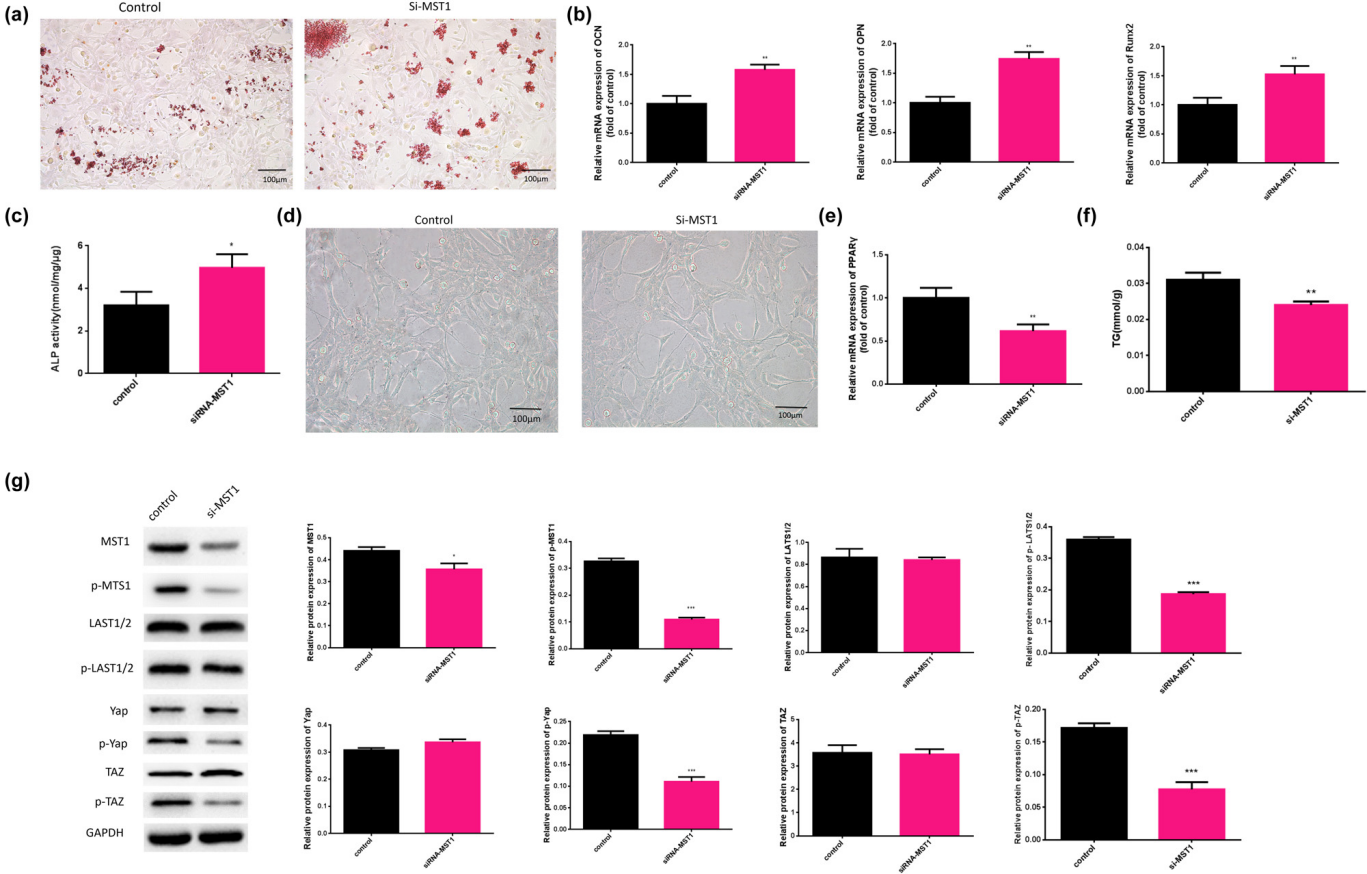
Downregulated MST1 alleviated the effects of osteogenesis and Hippo signaling and enhanced adipogenesis in BMSCs. (a) The calcium nodule form in BMSCs was detected by Alizarin Red S staining analysis. (b) The mRNA expression levels of OCN, OPN, and Runx2 in BMSCs treated with MPS were evaluated by qRT-PCR. (c) The ALP activity in MPS conditions was detected by ALP activity assay. (d) Oil droplets in BMSCs were observed using Oil Red O staining (×100). (e) The mRNA expression of PPARγ was evaluated by qRT-PCR. (b) The content of TG was measured in BMSCs. (f) The content of TG was measured in BMSCs. (g) The protein expression levels of MST1, p-MST1, Yap, p-Yap, LATS1/2, p-LATS1/2, TAZ, and p-TAZ were detected by western blot. *p < 0.05, **p < 0.01, ***p < 0.001. The experiment was repeated three times.

To verify our hypothesis, we used MST1-siRNA-treated BMSCs to observe the changes in osteogenic differentiation, adipogenic differentiation abilities, and Hippo signaling. The group treated with 10^−7^ mol/L MPS was regarded as the control. We repeated the above experiments to verify the effect of MST1-siRNA on BMSC osteogenic and adipogenic abilities and Hippo signaling pathway. The results showed that MST1-siRNA promoted BMSC osteogenic differentiation and increased the mRNA expression levels of Runx2, OPN, and OCN, with increasing ALP activity (Figure 5a–c). Meanwhile, MST1-siRNA significantly reduced the number of droplets, the mRNA expression of PPARγ, and the TG content (Figure 5d–f). In addition, the expression level of protein related to the Hippo signaling pathway was significantly reduced under si-MST1 treatment (Figure 5g).

## 4 Discussion

Necrosis of the femoral head due to pathological changes can result in joint surface collapse and the restriction of joint activity. A number of mechanisms for demonstrating the pathogenesis of steroid-based femoral head necrosis have been identified, including osteogenic differentiation balance disorder [14], fat embolism [15], cycle-blockade [16], cell apoptosis [17], and functional disorder [18]. In this study, we demonstrate that the abnormal control of osteogenesis and adipogenic differentiation in BMSCs plays an important role in the occurrence and development of GC-ONFH. Additionally, the impact of Hippo signaling on GC-ONFH progression was explored, and its potential mechanism was demonstrated.

Although the clinical overdose therapy of GC is related to the occurrence of ONFH, the exact mechanisms are still unclear. In general, bone marrow necrosis was found in GC-ONFH model with increased adipose tissue [19]. Additionally, numerous studies have demonstrated that excessive use of GC can lead to decreased osteogenic differentiation and increased adipogenesis of BMSCs [20,21]. Meanwhile, some reports suggest that BMSCs in GC-ONFH cases show decreased proliferation activity and reduced osteogenic differentiation ability [22]. In brief, these results indicated that GC-ONFH was involved in both BMSC-defective osteogenic and adipogenic differentiation. In our study, we found that BMSCs treated with MPS showed abnormal osteogenic differentiation and adipogenic differentiation, which was consistent with the results of previous studies.

The Hippo signaling pathway is composed of a group of conserved kinases [6]. Previous studies have reported that when Hippo signaling is activated, phosphorylated YAP is replaced in the cytoplasm to promote degradation, while when Hippo signaling is inactivated, nonphosphorylated YAP is transferred to the nucleus to induce the transcriptional activity of genes involved in cell growth [23,24]. DuPont et al. believe that MST1/2 and Lats1/2, as upstream regulatory proteins of TAZ/YAP, balance the phosphorylation of TAZ/YAP during the normal metabolism of Hippo signaling [25]. Runx2 is a transcription factor that stimulates osteogenesis and can bind to the WW domain of TAZ [26]. A prior study suggested that TAZ, a downstream effector molecule of Hippo signaling, could promote the transcription of genes downstream of Runx2 and the expression of osteoblast marker genes ALP, OCN, and OPN [27]. Our results confirmed that the Hippo pathway was activated in BMSCs under MPS treatment and that osteogenesis was increased with Runx2, ALP, OCN, and OPN activation. In recent years, it has been reported that MST1, as the upstream gene of the Hippo signaling pathway, is widely involved in regulating cell differentiation, cell homeostasis, autophagy, and other core proteins regulating Hippo signaling through phosphorylation [28]. Therefore, MST1 is considered to be an important indicator of Hippo signaling pathway activation. As expected, our results also showed that siRNA-MST1 inhibited the activity of Hippo signaling and promoted the osteogenic effects of BMSCs, indicating that osteogenesis in BMSCs might be regulated by Hippo signaling. From an earlier study, BMSCs in GC-ONFH cases showed decreased proliferation activity and reduced osteogenic differentiation ability [21]. In a study by Hong, TAZ was found bind to the adipogenic transcription factor PPARγ and inhibit the transcription of downstream target genes [26]. In our study, we also found that the Hippo pathway was activated in BMSCs under MPS conditions, accompanied by the inhibition of adipogenic differentiation and decreased expression of PPARγ, which can be reversed by siRNA-MST1. Collectively, based on the expression of proteins related to adipogenic and osteogenic differentiation and Hippo signaling-related protein expression, we hypothesized that Hippo signaling is an important signaling pathway involved in the differentiation of adipogenesis and osteogenesis in the pathological process of GC-ONFH.

In conclusion, for the first time, we found that the Hippo signaling pathway increased significantly in BMSCs under MPS conditions, which was consistent with the expression of the adipogenic differentiation-related PPARγ protein and related proteins of osteogenic differentiation, including Runx2, OPN, and OCN. In addition, si-MST1 decreased the adipogenic differentiation of BMSCs and promoted osteogenic differentiation by inhibiting Hippo signaling. Based on the above analysis, we predict that the Hippo signal may act as an important signaling pathway in the regulation of adipogenic and osteogenic differentiation in the presence of GC-ONFH.

Our study had some limitations. First, many signaling pathways regulate BMSCs; however, our present study only investigated the Hippo signaling pathway. We should provide further evidence by analyzing TCGA data to provide more mechanistic evidence as a next step. Second, two or more siRNAs are generally selected to ensure experimental quality, but given the limited experimental time of this study, there was not enough time to supplement the finding with another siRNA experiment. We plan to supplement two or three siRNAs in a subsequent study as a next step. Third, the effects of GC treatment on other Hippo-related signaling pathways downstream of BMSCs were not assessed. In the present study, only osteogenesis and adipogenic differentiation were applied, which were based on data from preliminary studies. Further studies are needed to elucidate the effects of GC treatment.

## Figures and Tables

**Figure A1:**
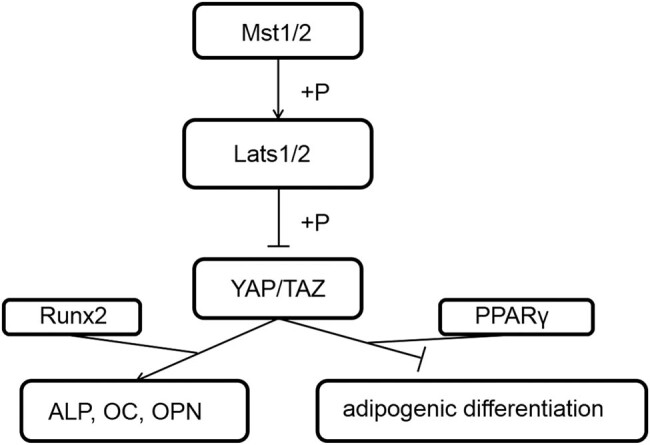
The Hippo signaling pathway schematic diagram.
